# Combining Inferential and Deductive Approaches to Estimate the Potential Geographical Range of the Invasive Plant Pathogen, *Phytophthora ramorum*

**DOI:** 10.1371/journal.pone.0063508

**Published:** 2013-05-07

**Authors:** Kylie B. Ireland, Giles E. St. J. Hardy, Darren J. Kriticos

**Affiliations:** 1 Cooperative Research Centre for National Plant Biosecurity, Canberra, Australian Capital Territory, Australia; 2 Centre for Phytophthora Science and Management, School of Veterinary and Life Sciences, Murdoch University, Perth, Western Australia, Australia; 3 Commonwealth Scientific and Industrial Research Organisation (CSIRO) Ecosystem Sciences, Canberra, Australian Capital Territory, Australia; Nanjing Agricultural University, China

## Abstract

*Phytophthora ramorum*, an invasive plant pathogen of unknown origin, causes considerable and widespread damage in plant industries and natural ecosystems of the USA and Europe. Estimating the potential geographical range of *P. ramorum* has been complicated by a lack of biological and geographical data with which to calibrate climatic models. Previous attempts to do so, using either invaded range data or surrogate species approaches, have delivered varying results. A simulation model was developed using CLIMEX to estimate the global climate suitability patterns for establishment of *P. ramorum*. Growth requirements and stress response parameters were derived from ecophysiological laboratory observations and site-level transmission and disease factors related to climate data in the field. Geographical distribution data from the USA (California and Oregon) and Norway were reserved from model-fitting and used to validate the models. The model suggests that the invasion of *P. ramorum* in both North America and Europe is still in its infancy and that it is presently occupying a small fraction of its potential range. *Phytophthora ramorum* appears to be climatically suited to large areas of Africa, Australasia and South America, where it could cause biodiversity and economic losses in plant industries and natural ecosystems with susceptible hosts if introduced.

## Introduction

*Phytophthora ramorum* is an invasive plant pathogen causing considerable and widespread damage in nurseries, gardens, natural woodland and plantation forest ecosystems of the USA and Europe [1], [2]. It is internationally recognized as a plant biosecurity threat in many regions. Australia, Canada, the Czech Republic, Mexico, New Zealand, South Korea and Taiwan have established specific quarantine policies and protocols to prevent the spread of contaminated plant materials from areas known to have the disease [3]. Outbreaks of the pathogen into areas of the west coast of the USA containing remnant native vegetation, particularly in central coastal California, have decimated populations of the keystone species tanoak (*Notholithocarpus densiflorus*) and coast live oak (*Quercus agrifolia*) [4], [5]. Similarly in England, *P. ramorum* has caused mortality of important ecological and commercial species such as *Vaccinium myrtillus* in heathlands [6] and Japanese larch (*Larix kaempferi*) in plantations [2]. While eradication efforts have been undertaken in natural areas of Oregon, USA [7] and parts of Europe [8], *P. ramorum* continues to invade new forest sites in these regions and in coastal California where eradication efforts have not been attempted.

While the geographical centre of origin for *P. ramorum* remains unknown, both molecular and biological evidence suggest that it is exotic to both North America and Europe. *Phytophthora ramorum* populations in Europe and North America are dominated by different mating types [9] and significant genotypic and phenotypic differences exist between these populations [10], [11], [12], [13]. At present, four distinct clonal lineages (NA1, NA2, EU1 and EU2) have been identified from North America and Europe [11], [14]. NA1, NA2 and EU1 are all found in North American nurseries, while NA1 is the only genotype present in Californian and Oregon forests [11]. In Europe, EU1 is the dominant genotype in nurseries and forests, while EU2 is a newly discovered genotype only known at present from infected *L. kaempferi* in Northern Ireland and Southern Scotland [14]. Although the disease emerged around the same time in nurseries in Europe and woodlands in California (the early to mid-1990s), molecular evidence suggests these lineages diverged at least 150 000 years ago and are most probably independent introductions from its native range [15]. The current geographical range of the pathogen in North American native vegetation ecosystems extends over 850 km from south of Big Sur in California to Curry County in Southwest Oregon [16]. The pathogen has also been recorded in streams as far north as King County in Washington state in the Pacific Northwest of the USA and associated with streams with inlet water from nurseries in Alabama, Georgia, Florida, Mississippi and North Carolina [17]. In Europe, natural outbreaks have largely been limited to southern England and south Wales in the UK [2], [18], with smaller outbreaks in public greens and woodlands of Belgium, Denmark, France, Germany, Ireland, Luxembourg, the Netherlands, Norway, Slovenia, Spain and Switzerland [8]. Infected nursery stock has been detected in 22 European countries, Canada and in numerous states in the USA where it predominates in the west coast states of Washington, Oregon and California [8], [19].

Movement of nursery stock has been highlighted as the primary factor spreading the pathogen both within the USA and globally [20]. This is of particular concern given that the geographical centre of origin for *P. ramorum* remains unknown, the trade in mature plants for horticulture and landscaping continues to grow [21] and biosecurity agencies rely on knowledge of the distribution of unwanted invasive alien organisms to ensure the application of effective quarantine regulations.

In order to justify both phytosanitary measures associated with international trade and domestic biosecurity actions aimed at slowing or preventing the spread of an established pest, under the International Standards for Phytosanitary Measures (ISPM), it is necessary to estimate the *endangered area* and the value of the impacts that might arise if the pest were to spread throughout that range [22]. Previously, a variety of models have been used to answer questions related to the probability and consequences of an invasion of *P. ramorum*. Techniques applied include niche modelling using programmes such as CLIMEX [23] and Genetic Algorithm for Rule-set Production (GARP) [24], as well as fine-scale tactical models and maps to address immediate management issues using support vector machines [25], rule-based expert elicitation approaches incorporating both climatic and host data [26], [27] and most recently, spatio-temporal, stochastic epidemiological modelling in combination with realistic geographical modelling to estimate spread [28]. Despite the importance of understanding the potential geographical range of *P. ramorum*, most studies have modelled its potential effects and spread where it already exists in the United States and Europe [8], [23], [25], [29], [30], [31], [32], [33]. Only two models have explored the potential for disease establishment and outbreaks globally [24], [27], and neither of these approaches addressed the question of the endangered area in terms of the ISPM framework.

While the various geographical models of *P. ramorum* were developed with the best available information at the time, many have failed to produce robust pest risk maps due to either a lack of suitable information to parameterise the model or the incorrect application or interpretation of a particular modelling method for simulating an emerging infectious disease. Distribution data used to inform models such as Guo et al. [25] and Kluza et al. [24] remain incomplete, as it is clear that the pathogen has not filled its ecological niche in its invaded range. In basing their projections on the known Californian distribution data at the time, these are best described as tactical models, estimating the invaded environment at that point in time, rather than the potential distribution of the pathogen. As a result, both of these models face challenges to the extrapolation of their outputs beyond that point in time and beyond the locations they are based upon.

CLIMEX [34], [35] and NAPPFAST [27] are software packages designed to deal with the piecemeal nature of information regarding invasive species. Both are capable of incorporating knowledge of an organism’s response to environmental variables, particularly climatic data, gleaned from direct observations or inferred from phenological observations. Both models can use information regarding the species’ potential to grow as a function of climatic factors to estimate the potential for the species to grow at any site for which suitable climatic data are available. Where NAPPFAST has a more detailed treatment of the potential for infection, CLIMEX is also able to use distribution data or experimental data to assess the potential for the species to survive inclement seasonal conditions. CLIMEX has been used previously to model potential distribution and relative disease risk of important plant pathogens on both continental [23], [36], [37] and global scales [38], [39].

Both the Venette and Cohen [23] CLIMEX and Magarey et al. [27] NAPPFAST models were based on the best ecophysiological data available at the time to define their parameters. The CLIMEX model [23] was built using some parameter values for *P. cinnamomi*, a congeneric species which is understood to be a species adapted to warmer climates [40], to define soil moisture and stress parameters for *P. ramorum*. Whilst informative at that time, laboratory and field-based studies have since revealed more information about the ecological and climatic factors necessary for the infection, transmission and persistence of *P. ramorum* [41], [42], [43], [44], [45], [46]. This new knowledge can now be used to inform an improved niche model specific to *P. ramorum*, and hence improve the understanding of the geographical risks posed by this organism.

The objective of this study was to estimate the global climate suitability patterns for the establishment of *P. ramorum* by developing a climatic niche model using the Compare Locations model in CLIMEX [34], [35]. Parameters were defined using revised growth and stress parameters for *P. ramorum*, based on the best available experimentally-derived ecophysiological responses and site-level phenological factors of transmission and disease persistence associated with climate data from the field. Independent distribution data from the USA (California and Oregon) and Norway were used to validate the model. The results of the model are discussed and related to quarantine and management implications for international plant biosecurity.

## Materials and Methods

The Compare Locations function in CLIMEX 3.0 [34] was used to develop a simulation model to estimate the climate suitability for the establishment of *P. ramorum* populations. The CliMond CM10_1975H_V1 interpolated climate surface [47] was used for all modelling. It is a fine scale (10 arc minute) dataset of long-term monthly climate means centred on 1975 for precipitation, maximum temperature, minimum temperature and relative humidity at 9 am and 3 pm. CLIMEX interpolates the monthly means to weekly values prior to calculating growth and stress indices.

The Compare Locations function in CLIMEX calculates an annual index of climatic suitability, the Ecoclimatic Index (EI), which reflects the combined potential for population growth during favourable periods and survival during stressful periods (Equation 1). The annual growth index (GI_A_) describes the potential for growth of the host and pathogen as a function of average weekly soil moisture (Moisture Index; MI) and temperature (Temperature Index; TI) during favourable conditions (Equation 2; Table 1; Weekly Thermo-hydrological Growth Index, TGI_W_ = TI x MI). Stress indices describing cold stress (CS), wet stress (WS), heat stress (HS), and dry stress (DS) (Table 1) and their interactions with one another can be used to describe the species response to climatically unfavourable conditions. The individual components of stress are combined into a stress index (SI) and a stress interaction index (SX) (Equations 3 and 4; CDX = Cold-Dry Stress, CWX = Cold-Wet Stress, HDX = Hot-Dry Stress and HWX = Hot-Wet Stress) [34].
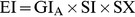
(1)

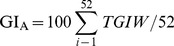
(2)

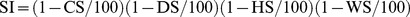
(3)

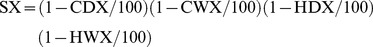
(4)


**Table 1:** CLIMEX parameter values used to model eco-climatic suitability of Phytophthora ramorum.

Parameter	Description	Value	
Temperature Index (TI)			
DV0	Lower temperature threshold for growth	0°C	
DV1	Lower optimum for growth	18°C	
DV2	Upper optimum for growth	22°C	
DV3	Upper temperature threshold for growth	30°C	
Moisture Index (MI)			
SM0	Lower soil moisture threshold for growth	0.2^a^	
SM1	Lower optimum for growth	0.7^a^	
SM2	Upper optimum for growth	1.3^a^	
SM3	Upper soil moisture threshold for growth	2.0^a^	
Cold stress (CS)			
TTCS	Temperature threshold for cold stress	−8°C	
THCS	Cold stress accumulation rate	−0.02 week^−1^	
Heat Stress (HS)			
TTHS	Temperature threshold for heat stress	31°C	
THHS	Heat stress accumulation rate	0.03 week^−1^	
Dry Stress (DS)			
SMDS	Soil moisture threshold for dry stress	0.2^a^	
HDS	Dry stress accumulation rate	−0.005 week^−1^	
Wet Stress (WS)			
SMWS	Soil moisture threshold for wet stress	2.0^a^	
HWS	Wet stress accumulation rate	0.002 week^−1^	

a Expressed as a proportion of soil moisture holding capacity, where 0 =  oven dry and 1 =  field capacity (saturation).

The EI ranges from 0 for locations at which the species is not able to persist to 100 at locations that are optimal for the species year round. To downplay the implied modelling precision of the percentile values of EI provided by CLIMEX, the EI was classified into four arbitrary classes: unsuitable (EI = 0), marginal (EI = 1–5), moderately favourable (EI = 6–25) and highly favourable (EI>25), as described by Kriticos et al. [48].

Phenological observations, and relevant laboratory and field-based biological information were used to inform the selection of relevant parameters for growth and stress of *P. ramorum* (Table 1). Data were combined for the three main *P. ramorum* genotypes, NA1, NA2 and EU1, to produce a composite model of the potential geographic range of the pathogen as insufficient data exists at this point in time to treat each lineage separately. The stress indices were fitted in such a way as to conform to the guidance of Kriticos et al. [49], so that the stresses and growth should not occur at the same time and hence the thresholds for stresses occur outside the limits for growth.

### Temperature Index

Temperature parameters were estimated from laboratory studies of the pathogen *in vitro* [41], [42], [45], [50], *in vivo* [51], [52] and from field-based studies of natural forest infections [53]. The lower temperature threshold for growth (DV0) was set to 0°C as infection and lesion growth can barely occur at this temperature [52], and below 0°C chlamydospore production and germination is impaired [45]. The lower and upper optimum temperatures for growth (DV1 and DV2) were set at 18 and 22°C, respectively, based on *in vitro* laboratory studies by Werres et al. [41], Tooley et al. [51] and Englander et al. [42] and optimum conditions for sporangia production and transmission under natural conditions [53]. The upper temperature threshold for growth (DV3) was set at 30°C based on the work of Werres et al. [41] and Brasier et al. [50]. Isolate growth and disease progression have not been observed above 30°C [50], [51], [53].

### Moisture Index

The soil moisture index in CLIMEX is used as a proxy for moisture availability, relying on observed correlations between soil moisture and relative humidity and/or rainfall [34], [38]. Moisture parameters for *P. ramorum*, not based on soil moisture, such as extended periods of leaf wetness (24 to 48 hours), high levels of rainfall and extended rainfall, have been associated with increased disease in the laboratory [51] and under natural field conditions [53]. These studies and those by Fichtner et al. [43] on the effect of soil drying on pathogen recovery, were used to infer and refine soil moisture parameter estimates. Extra drying of the substrate for the lower limit for growth (SM0), which was set to 0.2, was allowed for (expressed as a proportion of soil moisture holding capacity, where 1 =  saturation and >1 indicates excess moisture, i.e. run-off). The lower soil moisture value for optimal population growth (SM1) was taken from Brasier and Scott [54], as modified by Sutherst et al. [55] and subsequently applied by Venette and Cohen [23] to *P. ramorum*. Following iterative sensitivity analyses, the upper soil moisture value for optimal population growth (SM3) from these models was reduced from 3 to a more realistic value of 2, in order to indicate that wet conditions favour *P. ramorum* transmission and infection, but recognising that it was possible to have excessive amounts of rainfall and soil moisture.

### Cold Stress

Cold stress parameters were based on the results of recovery of chlamydospores (an asexual survival structure) at extreme cold temperatures [45], [52]. Tooley et al. [45] observed reduced recovery of free chlamydospores after exposure to temperatures of 0°C for 24 hours and little or no recovery at −10°C and −20°C, while Turner et al. [52] found they were able to survive at −2°C for up to four hours in the laboratory. Hyphal colonies on the other hand have been shown to have no reduction in recovery after exposure to −5°C for 24 hours, but reductions at or below −10°C [56]. Despite the direct effects of cold on the pathogen, chlamydospores were able to survive at least one week in infected leaf tissue after exposure to a continuous −10°C, indicating that survival in infected plant tissue provides a more robust method of pathogen survival during cold periods [45]. Turner et al. [52] also found that chlamydospores inside infected leaf tissue were able to survive mild winters with minimum temperatures of −9°C over 16 weeks under field conditions in the UK. At least 50% of chlamydospores survived for 16 weeks in the study by Turner et al. [52], with 80% chlamydospore survival in leaves buried 5 cm beneath the soil surface. In CLIMEX, the threshold cold stress due to damaging cold temperatures (TTCS) was set to begin accumulating at −8°C, at a slow enough rate (THCS = .−0.02 week^−1^) to allow for survival for at least two months at −10°C. This accumulation rate was chosen to incorporate survival in the coldest location where the pathogen is known to survive winter, in naturally infected nursery plants kept outside in the south of Finland (A. Rytkőnen, Finnish Forest Research Institute, pers. comm.)

### Heat Stress

Heat stress indices were fitted based on experiments that indicated that isolate growth and disease on plant material was impaired at temperatures above 30°C [41], [45], [50], [51], [53]. Brasier et al. [50] found that 37% of EU1 isolates and 80% of NA1 isolates grew at 30°C, but that neither lineage of isolates grew at 31°C. Similarly, Tooley et al. [45] found that chlamydospores of *P. ramorum* held in moist sand showed a high rate of recovery at 30°C, but at 35°C recovery declined steadily with time, and over a seven-day period there was no recovery of the pathogen at 40°C. Likewise, when Tooley et al. [45] tested recovery of *P. ramorum* from infected *Rhododendron* tissue they found high recoveries at 20 and 30°C after seven days, but at a constant 35°C recoveries declined within two days, and there was no recovery by four days. However, Tooley et al. [45] also found that *P. ramorum* was able to survive several weeks of maximum temperatures of 31°C and 32°C in a variable temperature growth chamber experiment, designed to represent temperature minima and maxima of an average summer in Lewisburg, Tennessee in the USA. Collectively, the results of these experiments indicate that heat stress (TTHS) for *P. ramorum* is likely to begin accumulating at or above approximately 31°C. The heat stress accumulation rate (THHS) was therefore set to accumulate at a rate of 0.03 week^−1^, to allow for a survival rate of at least 50% of *P. ramorum* under the conditions described by Tooley et al. [45], as those representative of a summer in Lewisburg, TN. A survival rate at or near 100%, as reported by Tooley et al. [45], was not calculated for as the methods used to break potential dormancy of *P. ramorum* in the Tooley et al. [45] study (where dry leaf disks were rehydrated for one hour prior to plating) are not considered likely to occur systematically and regularly under natural field conditions.

### Dry Stress

*Phytophthora ramorum* has been shown to be sensitive to drought, as highlighted by studies showing that free sporangia and chlamydospores were killed by drying at 30% relative humidity at room temperature for 30 minutes [57] and relative humidity below 50% significantly affected growth and germ tube elongation of zoospores [58]. However, sporangia have been recorded to survive up to six hours in moisture free conditions [52] and studies indicate that the pathogen can survive temperature and moisture stresses much more effectively within infected plant tissue [46]. Dry stress parameters were altered to begin accumulating below the lower soil moisture threshold for growth (SM0 = 0.2). The dry stress accumulation rate (HDS = −0.005 week^−1^) was selected to reflect the pathogen’s apparent sensitivity to drought.

### Wet Stress

Wet stress (SMWS) was set to occur when the soil moisture exceeds SM3 (2), with a relatively low stress accumulation rate (0.002 week^−1^).

### Model Run and Validation

The model was run on a world-wide scale using the CliMond historical climate data [47]. Geographical distribution data (both stream bait and infected tree confirmed positives) from California and Oregon [16] and Norway [59] were used to validate the model. The locations of ten positive waterways detected in the 2010 National *P. ramorum* Early Detection Survey of Forests [60] in Alabama, Georgia, Florida, Mississippi, North Carolina and Washington were also assessed in relation to the risk areas identified by the model.

## Results

### Model Fit and Projections

**Figure 1:**
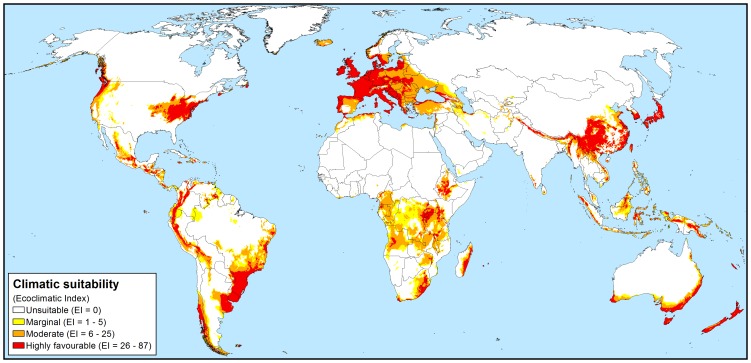
Global eco-climatic suitability for *Phytophthora ramorum* under the 1961–1990 climate normals, as modelled using CLIMEX.

**Figure 2:**
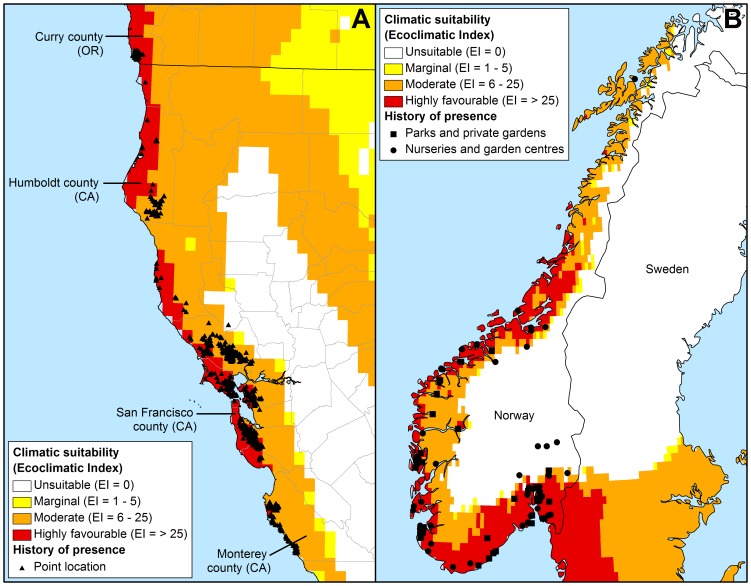
Known distribution of *Phytophthora ramorum* in northern California and Oregon, USA (a) and Norway (b) and eco-climatic suitability. Projected using the 1961–1990 climate normals, as modelled using CLIMEX.

The majority of Mediterranean and maritime temperate climates were projected to be favourable for *P. ramorum* and the model indicates that the pathogen could extend into some continental climates with warm or cool summers (e.g. in the western USA and south-eastern Canada) as well as some sub-tropical climates, such as Virginia and North Carolina in the USA and coastal northern New South Wales and southeast Queensland in Australia (Fig. 1). The modelled climate suitability fits the known occurrences within California and Norway and, as might be expected for a new invader, extends significantly beyond the known current distribution (Fig. 2).

### North America

**Figure 3:**
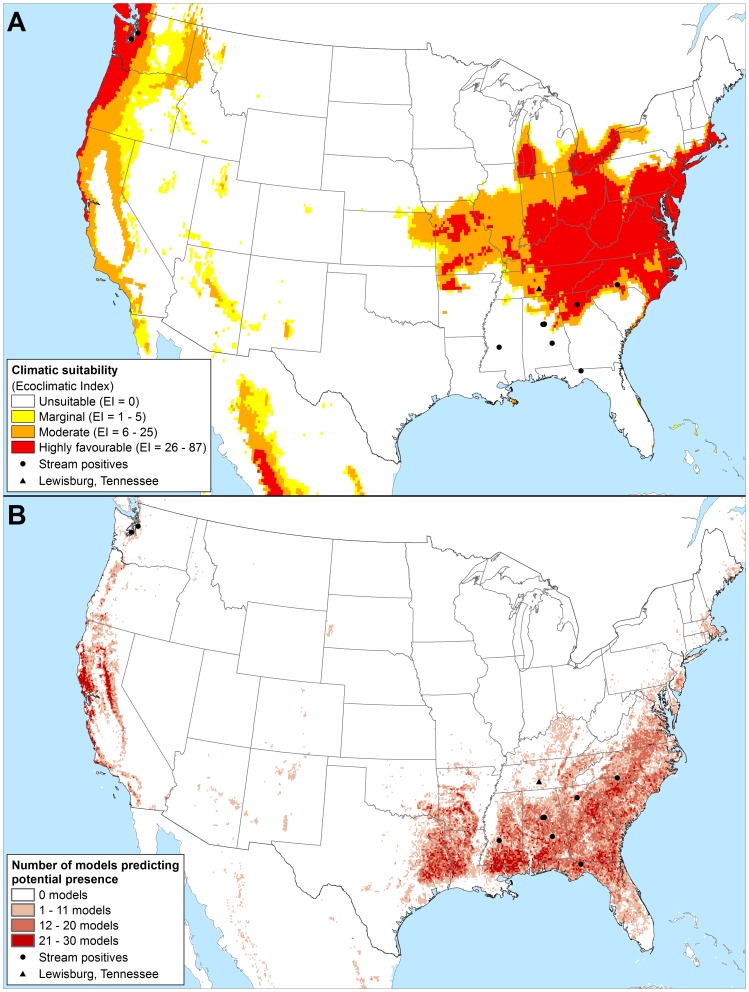
*Phytophthora ramorum* positive streams and models of potential range of the pathogen in the USA. Stream positive detections in the eastern USA and Washington state are those known at July 2010 [60]. As modelled using CLIMEX and the composite model parameters of our model under 1961–1990 climate normals (a); and as generated using a Genetic Algorithm for Rule-set Production (GARP) based on known Californian occurrences and three ecological datasets by Kluza et al. [24] (b).

The model suggests that the invasion of *P. ramorum* in North America is still in its infancy and it is presently occupying a small fraction of its available range (Figs. 2a and 3). All but one of the known positive detections (2696 total) of the pathogen in California and Oregon from the SODMAP data set [16] fell into the moderate to highly favourable risk areas (Fig. 2a). This one detection, NA-1, associated with infected *Umbellularia californica* trees, was located in Lake county, within 10 km of areas defined as being of moderate risk (Fig. 2a) and was a confirmed positive detection by PCR in 2006 [16].

Coastal areas with a Mediterranean climate along the west coast of the USA, extending into coastal areas of British Columbia (Canada), and continental areas of the East Coast, encompassing most of the Appalachians and extending into the Great Lakes region, appear suitable. Stream-associated nursery finds in Georgia and North Carolina fell into areas modelled as having highly favourable climates for pathogen establishment and survival, while those in Alabama, Florida and Mississippi fell into areas modelled as climatically unsuitable for persistence due to excessive heat stress (Fig. 3a; Supplementary Fig. S1). This does not preclude the species from epidemics in these areas, but indicates that populations are unlikely to be able to over-summer there. This is apparent in the high GI values throughout the south-eastern USA (Supplementary Fig. S2).

Within Central America and Mexico, areas classified as favourable were predominantly located at high elevations along the central mountain ranges. Large areas were modelled as climatically unsuitable for the establishment and persistence of *P. ramorum*, primarily due to a lack of adequate moisture for growth throughout the continental USA as well as being limited by temperature extremes in the far north and in the south toward the equator (Supplementary Figs. S1 and S3–S5).

### Europe

**Figure 4:**
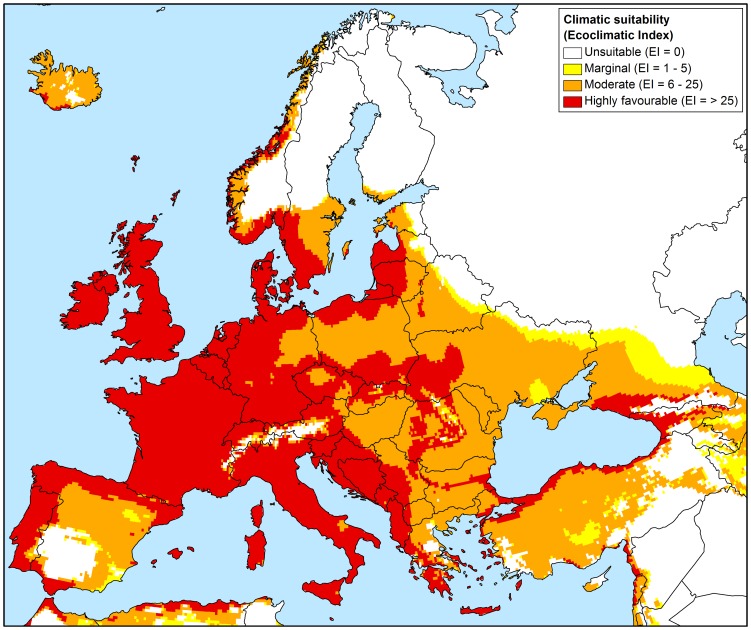
Known European distribution and eco-climatic suitability for *Phytophthora ramorum* **.** Projected using the 1961–1990 climate normals, as modelled using CLIMEX.

In Europe, it would appear that approximately half of the climatically favourable countries have already been invaded (22/45), predominantly within nurseries, though there is still considerable scope for further international spread and within country range expansion (Fig. 4). According to our model, all known countries in Europe where the pathogen has been detected have regions of moderate to highly favourable climate suitability for *P. ramorum* establishment. In Norway, all known places where the pathogen exists in parks and private gardens (59) and the majority (122/137) of locations of infected nursery and garden centres fell into areas with moderate to highly favourable climate (Fig. 2b). Almost all of Western Europe, apart from some high altitude areas in the Swiss Alps and the Carpathian Mountains in Romania (too cold; Supplementary Fig. S5) and southern regions of the Iberian Peninsula (too hot; Supplementary Fig. S1) were projected to have moderate to highly favourable climates for *P. ramorum* (Fig. 4). In Scandinavia, only the most southern coastal regions of Norway and Sweden and a small coastal port area of Finland were considered to be climatically favourable for the establishment and persistence of *P. ramorum* under historical climates. The majority of the Russian Federation and north-eastern regions of Belarus, Estonia, Latvia and Ukraine appear to be climatically unsuitable, due primarily to extreme cold weather conditions and inadequate soil moisture (Supplementary Figs. S4 and S5).

### Asia

**Figure 5:**
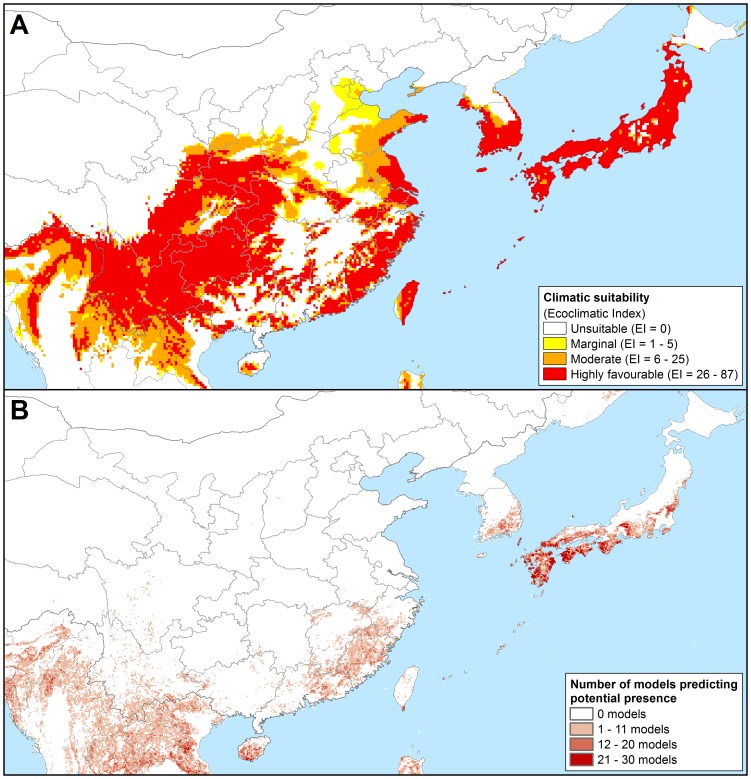
Projected potential distribution of *Phytophthora ramorum* in Eastern Asia using two different niche-models. As modelled using CLIMEX and the composite model parameters of our model under 1961–1990 climate normals (a); and as generated using a Genetic Algorithm for Rule-set Production (GARP) based on known Californian occurrences and three ecological datasets by Kluza et al. [24] (b).

Throughout Asia *P. ramorum* is projected to be primarily restricted to the humid sub-tropics (Figs. 1 and 5a), with some areas classed as having a continental climate with warm summers projected as favourable in China i.e., the central south eastern provinces of Yunnan and Sichuan, extending west across the Himalayas. Much of northern Asia, including Mongolia, western China and the Russian Federation appear too cold for *P. ramorum* (Supplementary Fig. S5). The Middle East is too hot and dry (Supplementary Figs. S1 and S4), and low-lying areas within tropical Asia are projected to be unsuitable for *P. ramorum* due to heat stress (Supplementary Fig. S1). In contrast, the majority of southern Japan and all of Taiwan were projected to be climatically moderate to highly favourable for *P. ramorum* (Figs. 1 and 5a).

### South America

The majority of South America was projected to be unsuitable for *P. ramorum* (Fig. 1), largely due to conditions not being conducive to pathogen growth (Supplementary Fig. S2) and heat stress (Supplementary Fig. S1). Moderate to highly favourable conditions occur at high altitudes in the Andes from northern Colombia to northern Argentina, and south of Concepción in Chile, where conditions are cooler. East of the Andes, the only region projected to be favourable was within a band on the East Coast, from Rio de Janeiro in Brazil to Bahia Blanca in Argentina.

### Africa

Moderate to highly favourable conditions for *P. ramorum* in Africa were largely restricted to elevated sites within Ethiopia, Kenya and along the borders of Uganda, Rwanda, Burundi and the Democratic Republic of Congo (Fig. 1). The majority of the continent was projected to be unsuitable for *P. ramorum* due to heat or dry stress (Supplementary Figs. S1 and S4). Central coastal subtropical areas of Angola, coastal subtropical areas of the East Coast and a small portion of the region of the Cape region of South Africa with a Mediterranean climate were also projected to have marginal to highly favourable climatic conditions for *P. ramorum* (Fig. 1). The model also projected moderate to highly favourable climates for the pathogen in eastern Madagascar.

### Australasia

In Australia, climatically favourable areas for *P. ramorum* were confined to the temperate moist periphery, predominantly in New South Wales, Victoria, Tasmania and south-west Western Australia, with coastal areas of the south east of Queensland and South Australia also projected as being favourable (Fig. 1). The pathogen was primarily restricted by hot, arid conditions in Australia (Supplementary Figs. S1 and S4). All of New Zealand was projected to have climates either moderate or highly favourable for *P. ramorum*, including the southern Alps, which extend above the tree-line (Fig. 1).

## Discussion

The CLIMEX model developed in this paper suggests that *P. ramorum* is presently occupying a small fraction of its available range in both North America and Europe. With continued global spread *P. ramorum* could potentially invade and establish in climatically favourable areas of Asia, Australasia, Africa and South America, where it could cause detrimental biodiversity loss and severe economic losses in plant industries and natural ecosystems with suitable hosts. It has been suggested by pathologists and modellers that the absence of *P. ramorum* from climatically favourable areas in North America is likely due to lack of historical opportunity, rather than intrinsic factors preventing its establishment [24], [25], [30], [33], [61], [62]. This hypothesis is supported by the model presented here, fuelling the debate as to why the pathogen has not established in the eastern USA. Early detection of *P. ramorum* in horticultural shipments to eastern USA, combined with effective quarantine measures on materials in infested areas and increased awareness of the disease [3], may have resulted in lack of establishment of the pathogen in these areas. Conversely, the pathogen may be in a lag phase of invasion in these areas or may have entered a state of equilibrium with the ecosystem and may not cause disease of epiphytotic proportions [63]. It has been suggested that *P. ramorum* may have been present in California for some years before the disease that it caused was noticed [1], so vigilance should be maintained when surveying in the eastern USA and maintaining and updating quarantine policies. Further research into this lag phase of *P. ramorum* invasion is crucial to understanding the aetiology of the pathogen’s disease cycle and could then be used to inform and formulate better risk models.

Given that all but one of the confirmed positive locations in California used to validate the model fell into areas that were moderate to highly favourable according to the model presented in this paper, as well as all known locations in Curry County, Oregon, where attempted eradication of the pathogen has been taking place [64]; our model suggests that surrounding areas identified as being climatically favourable areas are at high risk. Similarly, our model suggests that native vegetation communities in the Appalachian Mountains, for which potentially susceptible species have been identified in laboratory studies [65], are also at risk. Promisingly, the majority of stream associated nursery finds in the south-eastern USA fell into areas that experience prolonged periods of temperatures that are apparently too hot for *P. ramorum* (Supplementary Fig. S1) and may therefore present a reduced risk to native ecosystems; unless infected nursery stock growing in a protected environment provides a recurrent source of inoculum during periods favourable for infection and population growth of *P. ramorum*.

While the stream positive detection originating from a nursery in Mississippi was associated with infected streamside vegetation in 2010 [60], it is unclear whether this association represents a consistent microclimate that allows summer temperatures to be moderated sufficiently for persistence, or whether it is simply an ephemeral population that established between Autumn and Spring of that year, when both the CLIMEX (Supplementary Fig. S2) and NAPPFAST [27] models indicate that conditions are likely to be favourable for population growth. Subsequent surveys in this area over the past three years have failed to detect infected streamside vegetation again, despite continued stream positive detections (Steven Oak, USDA Forest Service, personal communication). While this may indicate an ephemeral streamside establishment of the pathogen associated with a suitable microclimate for that particular point in time, it may also simply be a reflection of sampling effort, chance infection and/or detection. This uncertainty illustrates one of the challenges of relying upon single field detections or laboratory observations recorded under constant or simplified diurnal temperature regimes to define stress parameters. Stress parameters are generally best-defined by fitting them to geographical distribution data based on consistent and/or well-designed sampling methodology, using mechanisms that are informed by laboratory experiments or theoretical expectations [66]. Irrespective of the explanation for this apparent discrepancy, one would expect that *P. ramorum* would be unlikely to establish a persistent population away from the streamside vegetation in this unsuitably hot region unless a favourable microclimate existed.

The Kluza et al. [24] model differed significantly from both the CLIMEX and NAPPFAST [27] model projections for the USA (Fig. 3; data not shown for NAPPFAST model). It is likely that the model of Kluza et al. [24] suffers from lack of sufficient input data, particularly given that it is unlikely that *P. ramorum* has reached its full expansion in the region upon which the model was based, in northern California. Using the Kluza et al. [24] model, vegetation surrounding the stream associated nursery finds in the south-eastern USA would be considered at a much greater risk of *P. ramorum* invasion (Fig. 3).

In Europe it would appear that approximately half of the climatically favourable countries (22/45) have recorded positive nursery finds, but few natural ecosystems, parks or private gardens have been invaded, leaving considerable scope for further within-country invasion. In Norway, which contains some of the most frigid conditions in which the pathogen may be able to survive, all natural infections in parks and private gardens and 89% of nurseries and garden centres which have been found to have positively infected stock [59] fell into areas modelled as being climatically suitable. Within-country invasion of areas outside of nurseries has been limited to only 11 European countries [8]. This may be a function of the pathogen not being spread to susceptible hosts in natural environments, lack of detection as a function of lack of surveillance, or may reflect the favourability of the artificial nursery environment, where temperatures may be regulated and plants are regularly watered, providing ideal conditions for the transmission of *Phytophthora* species. This lack of infestation in natural areas or gardens of climatically favourable countries may also be due to the lag phase of the pathogen invasion [63], as it was only discovered in the mid-1990s, and is likely to be at the beginning of its invasive spread. Therefore, continued and regular surveys for the pathogen outside the nursery environment may be warranted in these countries. The recent rapid spread of *P. ramorum* on *L. kaempferi* [18] in the UK highlights the potential for rapid spread elsewhere in climatically favourable regions.

Examination of the stress indices used in the models indicated that the distribution of the pathogen is largely restricted by extreme hot and dry conditions in the mid-latitudes, and cold stress in the high northern latitudes (Supplementary Figs. S1, S4 and S5). Nowhere in the southern hemisphere (excluding Antarctica) appears to be too cold for *P. ramorum*. Our models suggest that warm summer conditions may not be severe enough to reduce the risk of the pathogen establishing and spreading throughout most of the Mediterranean. It should be noted that the experimental data which underpin the selection of the heat stress parameters for *P. ramorum* in our model [45], [50], [51] are far from conclusive at this point in time, rarely exploring the conditions to break any potential dormancy of the pathogen. Our methodology and results reflect the best reasonable inference which could be derived at this point in time. Further experiments exploring the survival of *P. ramorum* propagules within smaller temperature increments (such as one degree increments between 30°C and 40°C, coupled with a range of dormancy breaking methods such as post-hydration of leaf discs or propagules [43]) would be invaluable in determining heat and heat/wet interaction stress parameters for the pathogen, and ultimately for informing the potential area of climatic suitability for *P. ramorum* in high risk areas such as the south-eastern USA.

Moisture, rainfall and days of consecutive rainfall are considered to play a crucial role in the development and spread of disease caused by *P. ramorum*, especially in years of high tree mortality, which historically follow very wet springs [53], [67]. This hypothesis has been supported by an experimental irrigation nursery study, which showed that the highest concentrations of infective propagules occurred when stream sampling was preceded by about two months with low minimum daily temperatures and by four days of high rainfall, indicating that cold wet conditions are highly conducive to disease development [44]. However, persistence and production of chlamydospores in forest and nursery soils and leaf litter over the summer contribute to the disease cycle by providing an inoculum reserve at the onset of the Autumn disease cycle [43], [46], [68], allowing the pathogen to survive otherwise non-conducive conditions for growth and survival. In the case of Greece, our model suggests that the pathogen would be able to survive the hot summer in this manner, contributing to the disease cycle at the onset of the rains in the winter, when conditions are ideal for *P. ramorum* transmission. However, should these moist conditions not occur following these initial dry conditions where the pathogen can survive, the risk of disease outbreaks and pathogen establishment may be negligible.

While the effects of weather cannot be explored in the CLIMEX Compare Locations function, further work utilising weather data from specific locations within CLIMEX and its sister program DYMEX (which models population dynamics) may be able to shed some light on the nature and influence of extreme weather events on *P. ramorum* disease incidence and severity. In this case, further questions as to the particular stages of the pathogen’s life cycle and their relationships to climatic variables and behaviour within populations would need to be addressed in much further detail to build such a model. At present, spread and potential establishment modelling utilising host data has been developed for California by Meentemeyer et al. [28], [30] to address some of the concerns associated with indicating where epidemics are most likely to occur in the near future. Models of this nature are valuable in a different way to our global establishment model, as in these regions the estimation of epidemic outbreaks, rather than simply the potential presence of the pathogen, is more important when making considered management decisions with finite financial resources.

Presently, CLIMEX is limited in not being able to use moisture parameters based upon relative humidity and leaf wetness, which are likely to be more influential to disease development than soil moisture alone, for aerial plant pathogens such as *P. ramorum*. Nevertheless, soil moisture is a relatively stable index that is very useful in defining the condition of the host plant and is influential in disease development, especially as it relates to dry stress and the permanent wilting point of the host plant [37], [38]. It is also regarded that soil moisture is a valid proxy for moisture availability when developing CLIMEX models for aerial pathogens [38]. Correlations between soil moisture and relative humidity and/or rainfall inform the selection of moisture related parameters when using the Compare Locations function in CLIMEX [34]. Detail on the interpretation of the relationship between rainfall and evaporation on the one hand and subsequent soil moisture parameter estimation on the other is outlined by Sutherst et al. [34], and it is recommended that new CLIMEX users make themselves familiar with the aforementioned relationships when estimating moisture based parameters.

Many opportunities exist in the future to further understand the underlying biology of *P. ramorum* and how this biology is associated with climatic and weather events. Study into changes of the potential range of *P. ramorum* under the effects of climate change would be valuable, especially as to how frequency of severe weather events such as particularly wet spring rains will influence disease spread, incidence and severity in areas where the pathogen is well established, such as California. As the pathogen becomes more widely established it may also be possible to apply methods of attaching severity ratings to the Ecoclimatic Index, as has been demonstrated as an effective modelling tool to plan future management in forestry by Pinkard et al. [37] for Tasmanian *Eucalyptus globulus* forests under the threat of Mycosphaerella leaf disease. This method also has applicability for understanding underlying mechanisms of pathogen behaviour and infective potential seasonally and across years [37].

This study adds to the body of work modelling the potential global distribution of *P. ramorum* [24], [69]. In particular, the model presented here builds upon the previous model developed by Venette and Cohen [23], who used CLIMEX to assess the potential climatic suitability for establishment of *P. ramorum* within the contiguous United States. Stress and soil moisture parameters of the surrogate *Phytophthora* species, *P. cinnamomi*, were replaced with those derived from the published literature on *P. ramorum*, much of which became available after the Venette and Cohen [23] paper was written. The modelled growth indices of our model and that of Venette and Cohen [23] are remarkably similar (Supplementary Fig. S6c and S6d), differing mostly in the ability to grow under dry conditions (data not shown). There were, however, significant differences in the potential range as indicated by the Ecoclimatic Indices (growth indices with stress indices applied; Supplementary Fig. S6a and S6b), with a significantly increased area at risk under our model in the west coast states and also the north eastern states, and a projected decreased risk in the southern (stream positive) states of Mississippi, Alabama and Georgia. This comparison highlights the importance of using and updating parameter sets providing the basis for models. Pest Risk Modelling has been described as the ‘art of the possible’ [70]. Risk models should be based on the best knowledge of an organism’s distribution and ecology at the time the model is developed, with scope to update and improve the model as new evidence becomes available.

Although our model clearly highlights areas at risk of invasion by *P. ramorum*, it should be noted that optimal climatic conditions for establishment do not necessarily translate into frequent or severe outbreaks. Not only must the pathogen be transported to suitable locations, and an infectious (sporulating) host be present in sufficient numbers to support infection in the long term at that location, but it must also be under the influence of local scale factors that may support severe outbreaks of the disease. Microclimatic factors play a big role in the presence of the disease in Northern California [30], and this may explain why one of the known presences used to validate the model in Northern California fell into an unsuitable area. Favourable microclimates for pathogen establishment and survival are unlikely to be apparent on the CliMond 10′ grid [66]. Notably, this positive detection of *P. ramorum* located in an area projected to be unsuitable by the model is located within 10 km of suitable climatic envelopes and associated with the highly favourable host, *U. californica*, lending weight to the idea that a suitable microclimate exists in this area. Nevertheless, climatic suitability is a necessary condition for any outbreak of the disease and the model projections provide a useful indication of potential outbreak severity. Given this, the risk maps provided in this paper could be used to provide guidance on areas to target for the early detection and monitoring of the pathogen globally.

Notwithstanding uncertainties about host range, no host implies no problem [71]. Furthermore, in the case of *P. ramorum*, no infectious host implies less of a problem, as rapid expansion of the pathogen’s range has been linked to “super-sporulating” hosts such as *U. californica* (California bay laurel) in California [72] and *L. kaempferi* (Japanese larch) in the United Kingdom [2], [73]. Potential host range studies of species from areas considered to be at risk of *P. ramorum* invasion have indicated that susceptible and infectious hosts exist on the east coast of the USA, Asia and Australasia [2], [74], [75], [76], [77], [78]. Knowledge of these hosts combined with knowledge of the potential geographical distribution is important when concentrating efforts for early detection of the pathogen, or when attempting to uncover the origins of invasive species such as *P. ramorum*. The origin of *P. ramorum* has been hypothesised by many to be in eastern Asia, based upon climatic variables [24] and origin of hosts such as *Rhododendron* [74]. Expeditions to regions of Yunnan Province in China, the centre of diversity for *Rhododendron* species, and Taiwan have not yet recovered *P. ramorum* [79], [80], [81]. Our results contrast very strongly with that of Kluza et al. [24] with regards to eastern Asia (Fig. 5). While both models agree that the Yunnan province in South-west China is climatically favourable, elsewhere the models disagree strongly. Where the model of Kluza et al. [24] indicates that the warmer humid tropical and sub-tropical climates are highly favourable for *P. ramorum* (e.g., Guangdong, Fujian, Zhejiang and Jiangxi provinces in China), our model indicates that it has a more temperate climatic preference (e.g. Guizhou, Hubei and Hunnan provinces in China). Perhaps targeting stream surveys for *P. ramorum* in the cooler regions of China, Taiwan (where the closely related *P.lateralis* has been discovered [81]) or Japan may be more fruitful.

Additional regions highlighted by the models, including temperate regions of China, elevated Andean locations in central South America and central Chile, the highly biodiverse Mediterranean region of the cape of South Africa, coastal Australia and the entirety of New Zealand, should be considered at risk of invasion by *P. ramorum*, or as potential origins of the pathogen, particularly where susceptible hosts naturally occur and/or are planted and traded. It is hoped the projections from this model will provide useful guidance on areas to target for early detection and monitoring of the pathogen, particularly in novel environments.

## Supporting Information

Figure S1 **Heat Stress (HS) for** ***Phytophthora ramorum*** **as modelled using CLIMEX with the CliMond dataset of historical climate normals centred on 1975.** Where HS = 0, heat does not limit the distribution of *P. ramorum* and where HS >0 heat stress is represented by a factor of 1000, with increasing limitation as HS increases.
(TIF)

Figure S2 **Annual Growth Index (GI; climatic suitability without stress) for** ***Phytophthora ramorum*** **as modelled using CLIMEX with the CliMond dataset of historical climate normals centred on 1975.** Climatic conditions are classified as being unfavourable for growth when GI = 0, marginally favourable when GI = 1–5, moderately favourable when GI = 6–25 and highly favourable when GI >25. The GI does not factor in climatic stress and therefore does not represent the potential distribution of *P. ramorum*, only growth during non-stressful periods of the year.
(TIF)

Figure S3 **Wet Stress (WS) for** ***Phytophthora ramorum*** **as modelled using CLIMEX with the CliMond dataset of historical climate normals centred on 1975.** Where WS = 0, soil moisture does not limit the distribution of *P. ramorum* and where WS >0 wet stress is represented by a factor of 1000, with increasing limitation as WS increases.
(TIF)

Figure S4 **Dry Stress (DS) for** ***Phytophthora ramorum*** **as modelled using CLIMEX with the CliMond dataset of historical climate normals centred on 1975.** Where DS = 0, soil dryness does not limit the distribution of *P. ramorum* and where DS >0 dry stress is represented by a factor of 1000, with increasing limitation as DS increases.
(TIF)

Figure S5 **Cold Stress (CS) for** ***Phytophthora ramorum*** **as modelled using CLIMEX with the CliMond dataset of historical climate normals centred on 1975.** Where CS = 0, cold does not limit the distribution of *P. ramorum* and where CS >0 cold stress is represented by a factor of 1000, with increasing limitation as CS increases.
(TIF)

Figure S6 **Ecoclimatic Index and suitability and Annual Growth Index for** ***Phytophthora ramorum*** **in the USA.** As modelled using the CLIMEX parameters of Venette et al. [23] (a and c) and our model (b and d), with the CliMond dataset of historical climate normals centred on 1975.
(TIF)
