# Supplementary figures and images for: Combining Inferential and Deductive Approaches to Estimate the Potential Geographical Range of the Invasive Plant Pathogen, Phytophthora ramorum
Source: PLoS One. 2013 May 7;8(5):e63508. doi: 10.1371/journal.pone.0063508 (PMC3646738; doi:10.1371/journal.pone.0063508)

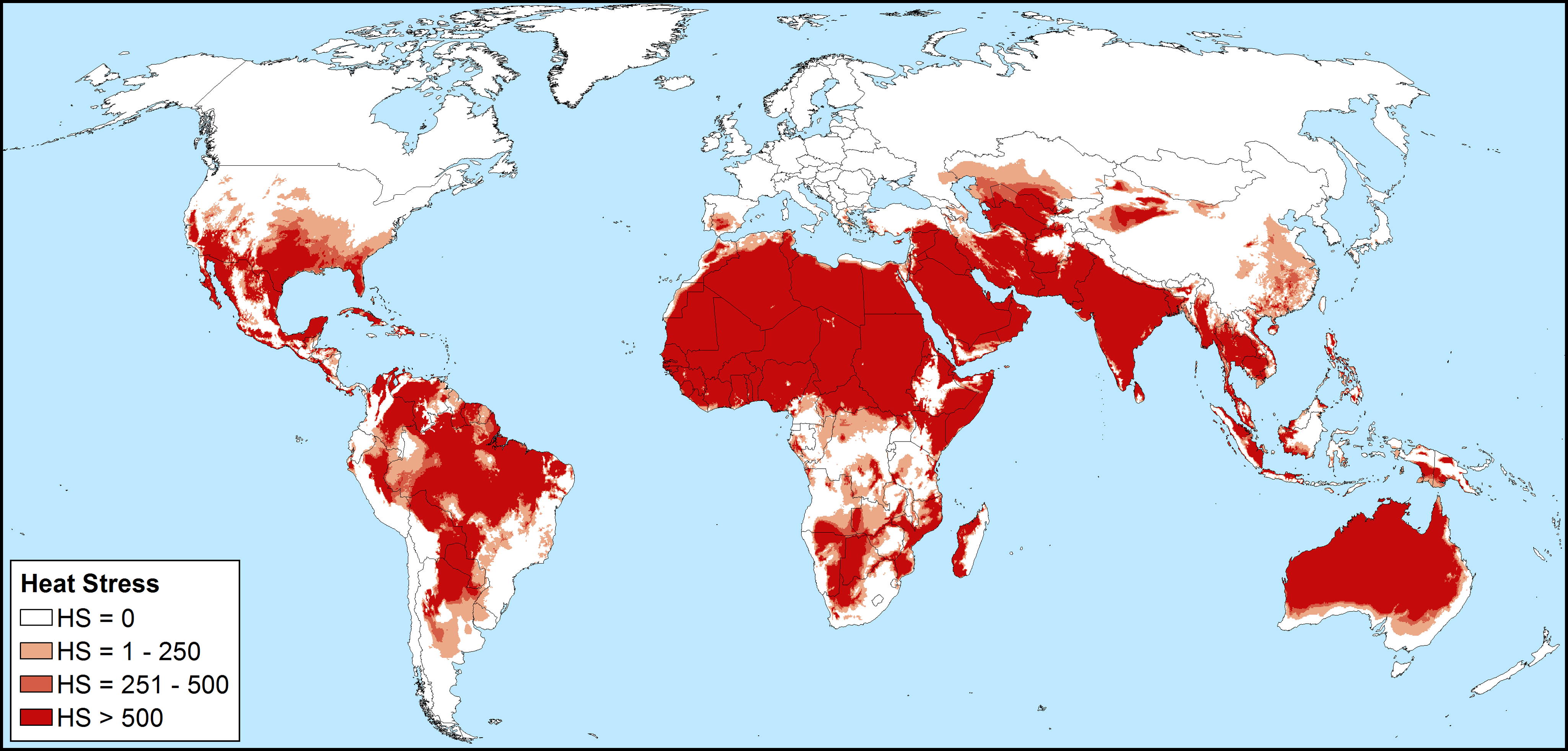

Supplement: Figure S1 — Heat Stress (HS) for Phytophthora ramorum as modelled using CLIMEX with the CliMond dataset of historical climate normals centred on 1975. Where HS = 0, heat does not limit the distribution of P. ramorum and where HS >0 heat stress is represented by a factor of 1000, with increasing limitation as HS increases. (TIF) [file pone.0063508.s001.tif]

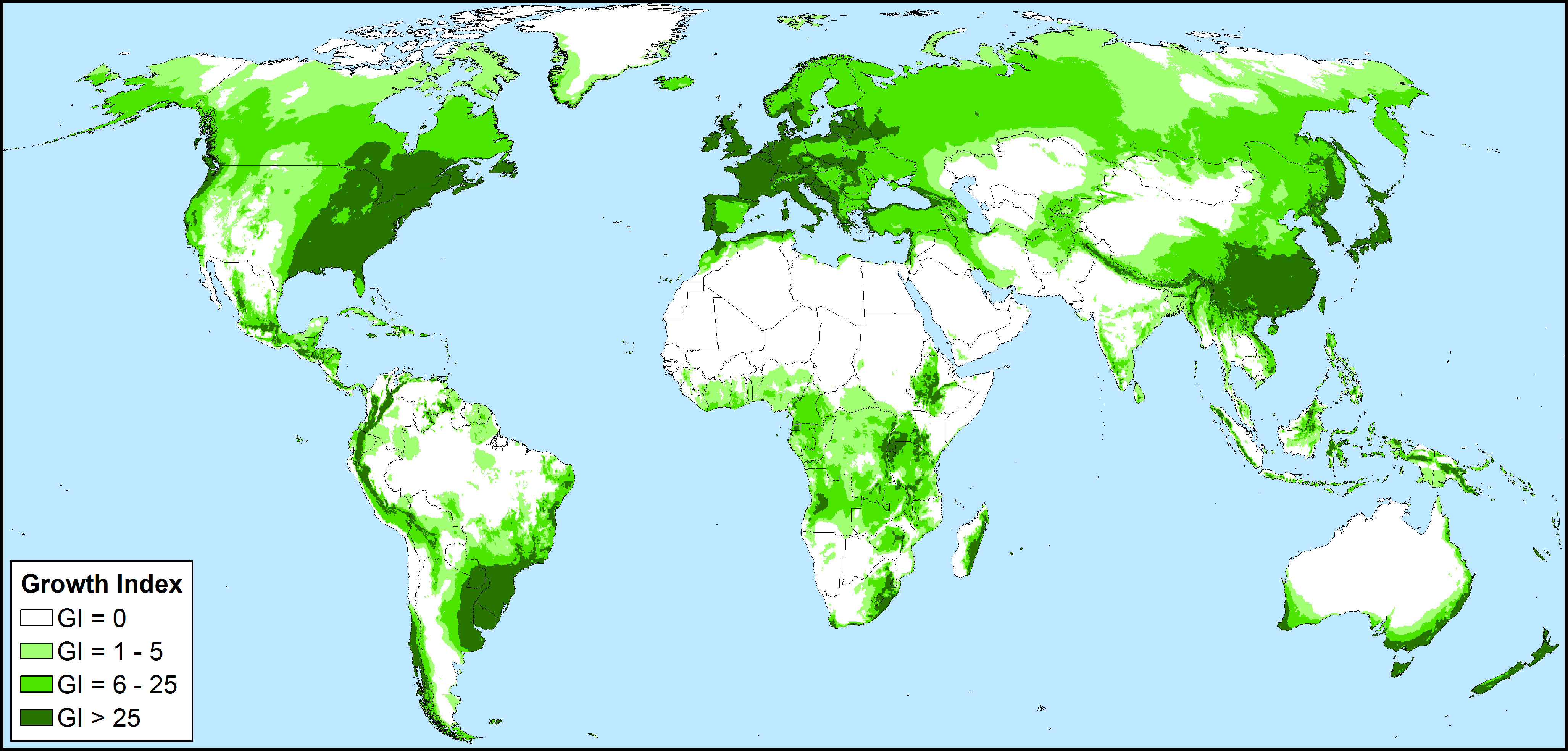

Supplement: Figure S2 — Annual Growth Index (GI; climatic suitability without stress) for Phytophthora ramorum as modelled using CLIMEX with the CliMond dataset of historical climate normals centred on 1975. Climatic conditions are classified as being unfavourable for growth when GI = 0, marginally favourable when GI = 1–5, moderately favourable when GI = 6–25 and highly favourable when GI >25. The GI does not factor in climatic stress and therefore does not represent the potential distribution of P. ramorum, only growth during non-stressful periods of the year. (TIF) [file pone.0063508.s002.tif]

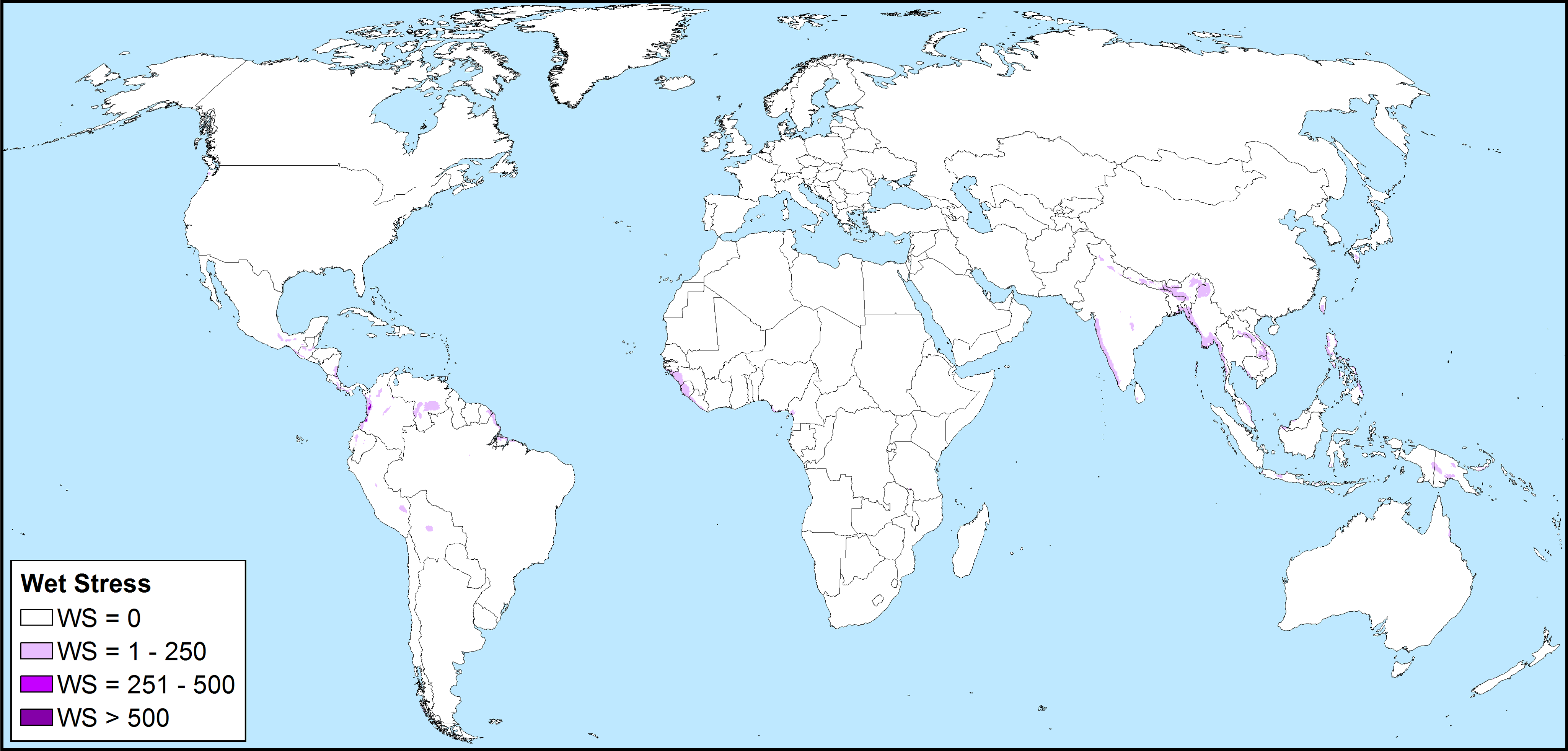

Supplement: Figure S3 — Wet Stress (WS) for Phytophthora ramorum as modelled using CLIMEX with the CliMond dataset of historical climate normals centred on 1975. Where WS = 0, soil moisture does not limit the distribution of P. ramorum and where WS >0 wet stress is represented by a factor of 1000, with increasing limitation as WS increases. (TIF) [file pone.0063508.s003.tif]

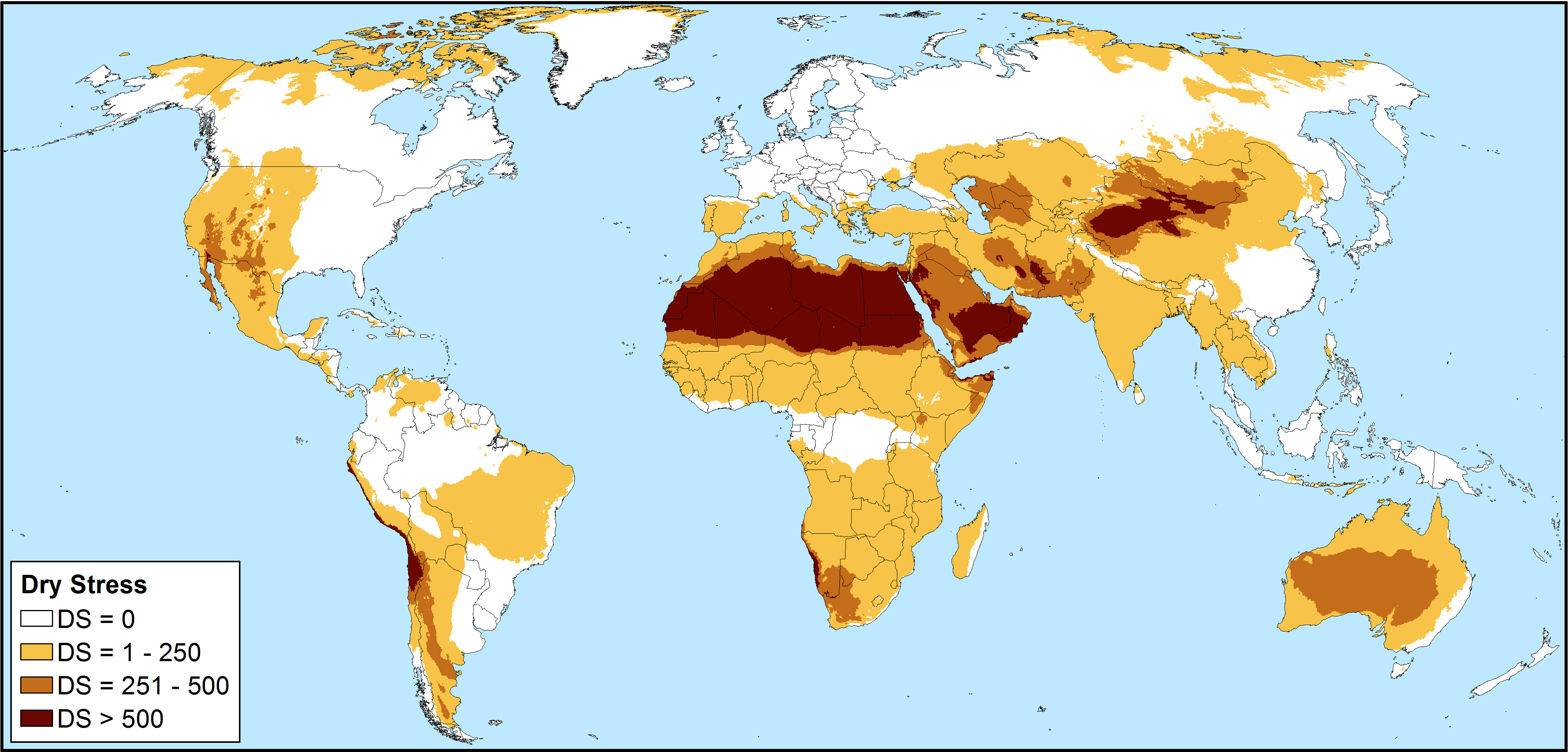

Supplement: Figure S4 — Dry Stress (DS) for Phytophthora ramorum as modelled using CLIMEX with the CliMond dataset of historical climate normals centred on 1975. Where DS = 0, soil dryness does not limit the distribution of P. ramorum and where DS >0 dry stress is represented by a factor of 1000, with increasing limitation as DS increases. (TIF) [file pone.0063508.s004.tif]

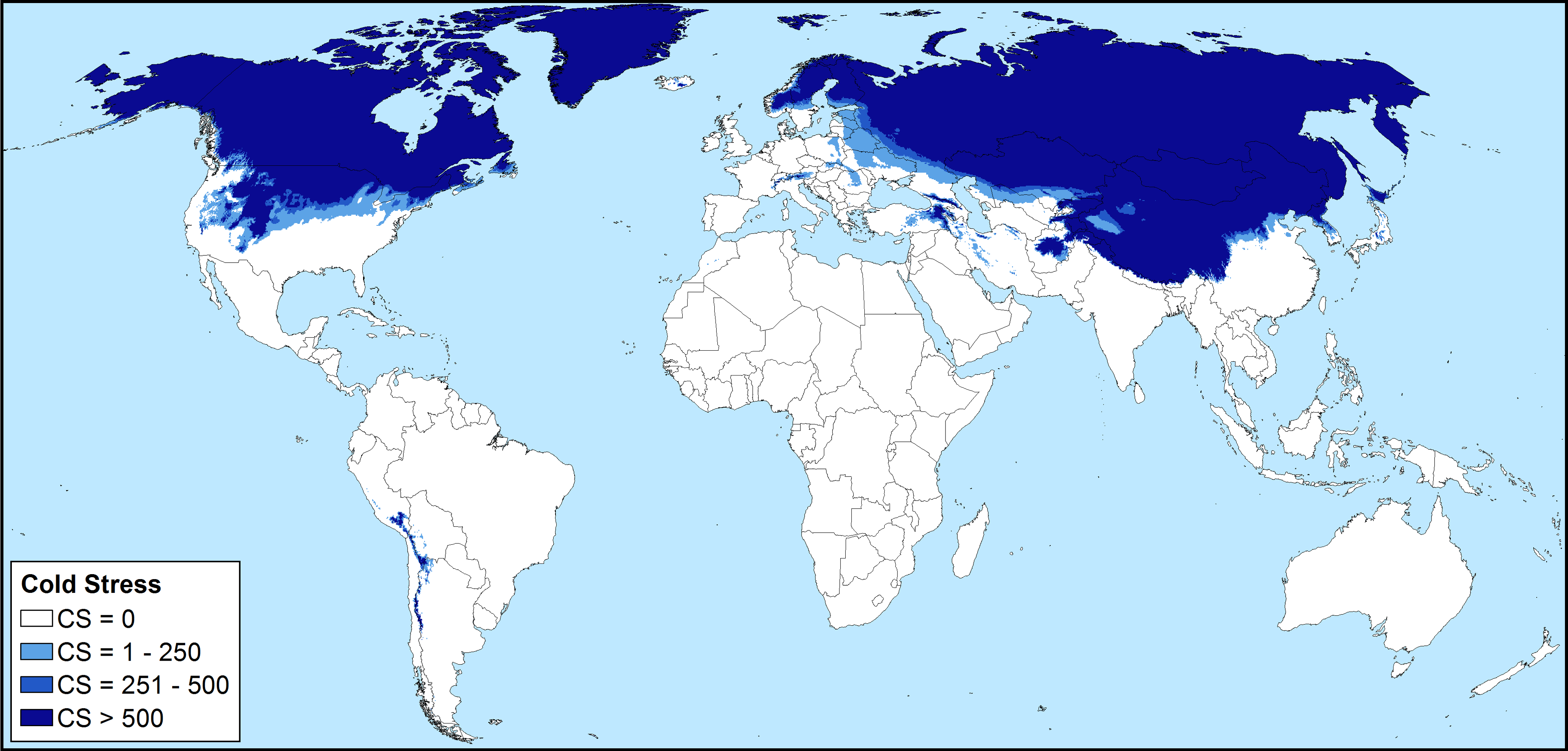

Supplement: Figure S5 — Cold Stress (CS) for Phytophthora ramorum as modelled using CLIMEX with the CliMond dataset of historical climate normals centred on 1975. Where CS = 0, cold does not limit the distribution of P. ramorum and where CS >0 cold stress is represented by a factor of 1000, with increasing limitation as CS increases. (TIF) [file pone.0063508.s005.tif]

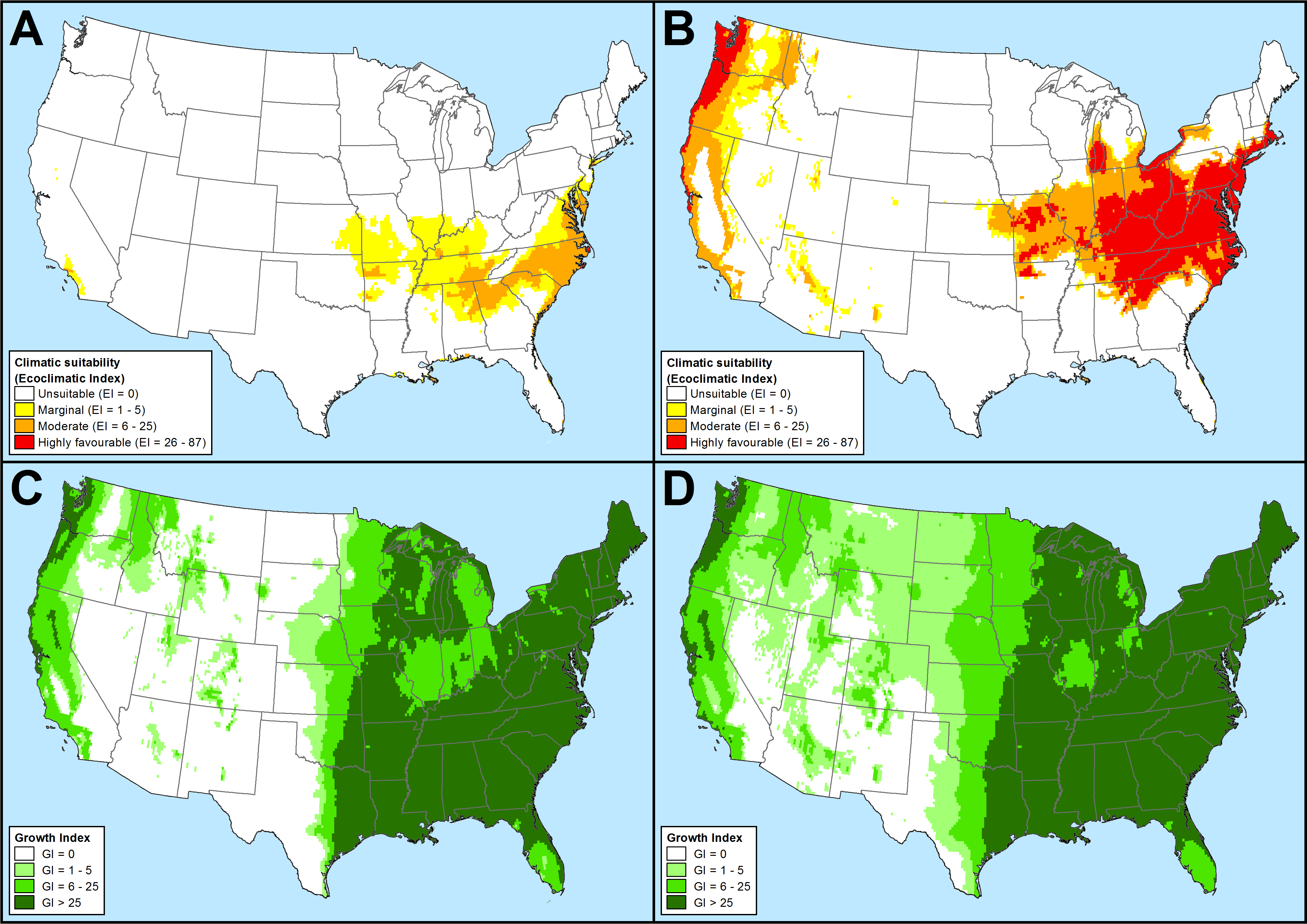

Supplement: Figure S6 — Ecoclimatic Index and suitability and Annual Growth Index for Phytophthora ramorum in the USA. As modelled using the CLIMEX parameters of Venette et al. [23] (a and c) and our model (b and d), with the CliMond dataset of historical climate normals centred on 1975. (TIF) [file pone.0063508.s006.tif]
